# One-year outcomes of mechanical thrombectomy and catheter-directed thrombolysis in acute pulmonary embolism

**DOI:** 10.1016/j.jvsv.2026.102636

**Published:** 2026-10-06

**Authors:** Bright Benfor, Pallavi Gorantla, Sri Achanta, Eric K. Peden

**Affiliations:** a Department of Cardiovascular Surgery, Houston Methodist Hospital, Houston, TX; b University of Texas at Austin, Austin, TX; c School of Engineering, Texas A&M University, College Station, TX

**Keywords:** Acute pulmonary embolism, Catheter-based therapy, Catheter-directed thrombolysis, Large-bore aspiration thrombectomy, Mechanical thrombectomy, Suction thrombectomy

## Abstract

**Background:**

Although anticoagulation remains the standard of care for acute pulmonary embolism (PE), mechanical thrombectomy (MT) and catheter-directed thrombolysis (CDT) have emerged as effective interventional options for rapid clot removal. However, their comparative outcomes remain incompletely defined. Hence, this study compared the outcomes of MT and CDT for acute PE at a tertiary referral center in the United States.

**Methods:**

We conducted a retrospective cohort study of all patients who underwent MT or CDT for acute PE across a single hospital system between January 2021 and April 2025. Patients were stratified into MT and CDT groups based on the treatment received. Baseline characteristics, procedural data, and clinical outcomes were analyzed. The primary outcome was 30-day mortality. The secondary outcomes included length of hospital stay, major adverse events, reintervention, and PE recurrence. Patients were followed up for up to 1 year or until death.

**Results:**

A total of 724 patients were included in this study: 569 (79%) underwent MT and 155 (21%) received CDT. The mean age was 63 ± 16 years, and 49% were female. Most patients (76%) were classified as intermediate-high risk, with a mean right ventricle to left ventricle ratio of 1.5 ± 0.5. Bilateral or saddle embolism was present in 93% of patients. Patients in the MT group were more likely to have a simplified PE severity index of >0 (73% vs 60%; *P* < .001). Age and the rates of cancer, chronic cardiopulmonary disease, and concurrent deep vein thrombosis were similar between the groups. The median procedure duration was longer for MT (66 minutes, interquartile range, 46-89) than for CDT (58 minutes, interquartile range, 40-77; *P* = .003). The median postoperative length of stay was 3 days in both groups. MT was associated with a higher incidence of intraoperative adverse cardiac events (2.5% vs 0%; *P* = .048), and CDT was associated with higher intensive care unit admission rates (83% vs 36%; *P* < .001). However, the 30-day mortality rate was similar between the groups (CDT: 2.6% vs MT: 4.6%; *P* = .27). Additionally, the 1-year incidence rates of chronic thromboembolic pulmonary hypertension (MT: 4.5% vs CDT: 3.4%; *P* = .69), PE recurrence (MT: 4.4% vs CDT: 2.5%; *P* = .31), and all-cause mortality (MT: 7.8% vs CDT: 5.8%; *P* = .41) were not statistically significantly different between the MT and CDT groups.

**Conclusions:**

In this retrospective cohort, MT and CDT for acute PE showed statistically comparable 30-day mortality rates and 1-year recurrence rates of chronic thromboembolic pulmonary hypertension and PE. MT was associated with a higher incidence of intraoperative cardiac complications, and CDT was associated with more frequent intensive care unit admission.

**Article Highlights**

• **Type of Research:** Single-hospital system retrospective cohort study
• **Key Findings:** In 724 patients undergoing catheter-based therapy for acute pulmonary embolism, both mechanical thrombectomy (n = 569) and catheter-directed thrombolysis (n = 155) had equivalent technical success rates (94.7% vs 93.6%; *P* = .57) and 30-day mortality rates (4.6% vs 2.6%; *P* = .270).
• **Take home Message:** These findings support the use of mechanical thrombectomy and catheter-directed thrombolysis as viable alternate treatment options within multidisciplinary pulmonary embolism response frameworks, with device selection best tailored to patient-specific risk profiles, anatomic considerations, and institutional expertise.

Acute pulmonary embolism (PE) is a major global health threat, and it remains one of the leading causes of cardiovascular death, with mortality rates continuing to increase over the past decade.^1^ The standard of care remains anticoagulation; however, catheter-directed therapies are recommended for patients with severe right ventricular (RV) strain.^1^, ^2^ Defining the optimal clinical approaches that provide effective and timely reperfusion without exposing the patient to hemorrhagic risk remains an area of active research.^3^, ^4^

Catheter-based therapies (CBTs), including mechanical thrombectomy (MT) and catheter-directed thrombolysis (CDT), are being adopted with increasing frequency in the management of acute PE, providing vascular surgeons and interventionists with tools to rapidly reduce the clot burden and improve symptoms through targeted, minimally invasive intervention.^5^

Comparative outcome data for CBT for acute PE remain limited, as most available studies have evaluated each modality individually rather than directly comparing MT and CDT. Existing studies have predominantly focused on short-term results, typically 7-day or 30-day outcomes.^6^, ^7^, ^8^ Additionally, they are mostly industry-sponsored controlled trials with strict exclusion criteria, making it difficult to extrapolate the results to real-world clinical practice. Long-term outcome data remain insufficient, emphasizing the need for real-world longitudinal studies to determine the relative effectiveness of each modality across diverse PE risk groups.^9^, ^10^, ^11^

This study compared the 1-year outcomes of CDT and MT in a single hospital system in Houston, Texas, USA. We hypothesize that both treatment modalities would yield similar results in patients with similar risk profiles.

The results obtained from the examination of the comparative outcomes of these techniques can inform patient selection and treatment optimization, thus guiding vascular surgeons or interventionists involved in PE response and CBT.

## Methods

### Study type and setting

We conducted a retrospective cohort study within a single, large hospital system in the Houston metropolitan area to compare the postoperative outcomes of MT and CDT for acute PE. These procedures were performed across seven hospitals within the system between 2021 and 2025. Institutional review board approval was obtained before study initiation.

### Patient selection and data collection

Consecutive adult patients presenting with acute PE who underwent MT or CDT during the study period were included. Patients were excluded if the procedure was aborted before the advancement of the thrombectomy or lysis catheter into the right heart or if key procedural and follow-up data were missing from medical records (n = 49). Demographic data, comorbidities, clinical characteristics, imaging findings, and perioperative variables that had been prospectively captured in electronic medical records were retrospectively extracted into a standardized database for analysis.

### Perioperative risk assessment and procedure details

All patients underwent thin-slice computed tomography pulmonary angiography and/or transthoracic echocardiography before intervention. RV strain was defined as an RV to left ventricular (LV) diameter ratio of ≥1.0 on computed tomography pulmonary angiography or as echocardiographic evidence of RV dysfunction.

Baseline clinical severity was assessed using the simplified pulmonary embolism severity index (sPESI).^12^ Patients were stratified into low, intermediate-low, intermediate-high, and high-risk categories according to the 2019 European Society of Cardiology (ESC) guidelines, which incorporate sPESI score, RV dysfunction, elevated cardiac biomarkers, and hemodynamic instability.^2^ Indications for CBT were determined by the institutional PE response team, which was vascular surgeon-led during the study period. The primary operators made the decision to intervene, which was generally based on the ESC classification of PE severity, clinical presentation, functional status, and life expectancy. Most procedures were performed by board-certified vascular surgeons in a hybrid operating suite under monitored anesthesia care with local anesthesia. A subset of procedures was performed in the cardiac catheterization laboratory by interventional cardiologists. The choice of the treatment and device was at the discretion of the primary surgeon or interventionist.

Ultrasound-guided percutaneous common femoral venous access was achieved in most patients, and baseline invasive pulmonary artery pressure was obtained in the majority of patients.

### Outcome measures and definition of variables

The primary exposure variable was the type of treatment, and the primary outcome was 30-day all-cause mortality. The secondary outcomes included technical success (successful device deployment with clot retrieval), postoperative length of stay (LOS), intensive care unit (ICU) utilization, in-hospital mortality, unplanned reinterventions, major adverse events, 1-year incidence of chronic thromboembolic pulmonary hypertension (CTEPH), recurrent PE, and overall survival. Patients were followed up for up to 1 year or until death.

### Statistical analysis

Continuous variables are presented as mean ± standard deviation or median with interquartile range (IQR), as appropriate. Categorical variables are expressed as frequencies and percentages. Patients were stratified into CDT and MT groups. Comparisons were performed using the χ^2^ or Fisher exact test for categorical variables and Student *t* test or Wilcoxon rank-sum test for continuous variables.

Subgroup analyses were performed according to the ESC risk category. Multivariable logistic regression was used to evaluate the association between the treatment type and 30-day mortality. Variables with *P* < .20 in the univariate analysis were entered into a backward stepwise model to identify the independent predictors of mortality in intermediate-high risk patients. All tests were two-tailed, with *P* ≤ .05 considered statistically significant. Analyses were conducted using STATA MP version 17.0 (StataCorp).

## Results

### Patient characteristics

A total of 724 patients were included, with a mean age of 63 ± 16 years; 49% were female, and 12% were aged ≥80 years. Specifically, 569 (79%) underwent MT, and 155 (21%) received CDT. The baseline demographics and comorbidities are summarized in Table I. Active smoking, prior PE, and concomitant deep vein thrombosis were present in 11.6%, 15.5%, and 73.2% of patients, respectively. Recent surgery (ie, within 3 months of presentation) was more frequent in the MT group (24.2% vs 16.2%; *P* = .036), and patients receiving MT were less likely to have a history of PE (14.1% vs 20.8%). Active malignancy was reported in 31% of patients undergoing MT, without statistically significant differences between the groups (MT: 31.4% vs CDT: 24.7%; *P* = 24.7). Most patients (76%) presented with intermediate-high risk PE. However, patients undergoing MT were more likely to present with high-risk PE (7.6% vs 3.9%; *P* = .014) and were more likely to have sPESI ≥ 1 (73.4% vs 60%). The mean right ventricle to left ventricle ratio (RV/LV) was 1.5± 0.5, without any significant difference between the groups.

**Table I:** Baseline characteristics of patients undergoing catheter-based therapies for acute pulmonary embolism between 2021 and 2025

Variable^a^	All N = 724	MT (n = 569)	CDT (n = 155)	*P* value
Demographics and comorbidities				
Age, years, mean ± SD	62.8 ± 15.5	63.0 ± 15.5	61.7 ± 15.9	.344
Age ≥80 years	11.7	12.3	9.7	0.368
Female sex	48.5	48.5	48.4	.979
Active smoker	11.6	10.7	14.8	.156
Recent trauma	4.3	4.5	3.9	.765
Recent surgery	22.5	24.2	16.2	.036
Active cancer	30.0	31.4	24.7	.105
Prior pulmonary embolism	15.5	14.1	20.8	.041
Chronic cardiopulmonary disease	17.3	17.2	17.4	.954
Concurrent/recent DVT	74.7	75.0	73.2	.643
Symptoms				
Syncope	20.0	19.6	21.4	.617
Preoperative cardiac arrest	1.8	2.1	0.7	.318
Chest pain	34.0	32.3	40.3	.064
Shortness of breath	80.4	77.9	89.6	.001
Vital signs				
SBP < 100 mmHg	10.1	10.4	9.0	.624
Heart rate > 110	36.5	37.8	31.6	.157
Pulse oximetry < 90%	11.1	10.7	12.3	.588
Labs/Imaging				
Elevated cardiac biomarkers	89.7	88.9	92.9	.148
Central thrombus burden	78.6	77.6	82.3	.415
RV/LV ratio (n = 679)	1.5 ± 0.5	1.5 ± 0.5	1.6 ± 0.4	.486
Right ventricle dysfunction	90.2	89.1	94.2	.059
Saddle/bilateral PE	94.1	93.9	95.2	.525
ESC risk severity				.014
High risk	6.8	7.6	3.9	
Intermediate-high	76.1	73.5	85.8	
Intermediate-low	15.2	16.7	9.7	
Low risk	1.9	2.3	0.7	
sPESI ≥ 1	70.5	73.4	60.0	.001

*CDT*, Catheter-directed thrombolysis; *DVT*, deep vein thrombosis; *MT*, mechanical thrombectomy; *PE*, pulmonary embolism; *sPESI*, simplified pulmonary embolism severity index; *RV/LV*, right ventricle to left ventricle ratio; *SBP*, systolic blood pressure; *SD*, standard deviation.

a Categorical data are expressed as %; continuous data are expressed mean ± SD.

### Procedural characteristics

The median procedure duration was longer in the MT group (66 [IQR, 46-89] vs 58 [IQR, 40-77] minutes; *P* = .003). High technical success rates were obtained in both groups (94.7% vs 93.6%; *P* = .57). Concomitant inferior vena cava filter placement occurred in 17% of patients, and such placement was more common in the MT group (21% vs 6.5%; *P* < .001). Similarly, simultaneous lower extremity venous thrombectomy was performed less frequently in patients undergoing MT (5.8% vs 0.0%; *P* = .002). Intraoperative cardiovascular collapse was more frequent in the MT group (2.5% vs 0%; *P* = .048) (Table II).

**Table II:** Intraoperative details and postoperative outcomes of patients undergoing catheter-based therapies for acute pulmonary embolism between 2021 and 2025

Variable^a^	MT (n = 569)	CDT (n = 155)	*P* value
Procedure duration, minutes	66 [46-89]	58 [40-77]	.003
Technical success	94.7	93.6	.57
Intraoperative cardiovascular collapse	2.5	0.0	.048
Postoperative ICU stay	36.4	83.0	<.001
Postop ICU stay duration, hours	0 [0-22.8]	24 [17-40]	<.001
Total postoperative LOS, days	3 [2-5]	3 [2-4]	.212
In-hospital mortality	3.2	0.7	.093
30-day major adverse event	8.8	6.5	.284
30-day mortality	4.6	2.6	.270
30-day reintervention	4.0	5.8	.343
1-year PE recurrence	4.4	2.5	.310
1-year CTEPH	4.5	3.4	.659

*CDT*, Catheter-directed thrombolysis; *CTEPH*, chronic thromboembolic pulmonary hypertension; *ICU*, intensive care unit; *LOS*, length of stay; *MT*, mechanical thrombectomy; *PE*, pulmonary embolism.

a Categorical data are expressed as %, continuous data are expressed as median [interquartile range].

### Clinical outcomes

The median postoperative LOS was 3 days (IQR, 2-5), with no significant difference between the groups. Postoperative ICU stay was more frequent in the CDT group (83% vs 36.4%; *P* < .001), with a median postoperative ICU LOS of 24 hours in the CDT group [IQR, 17-40 hours] vs 0 hour [IQR, 0-23 hours] in the MT group (*P* < .001). In-hospital mortality tended to be higher in the MT group (3.2% vs 0.7%; *P* = .093), but the finding did not achieve statistical significance. The 30-day major adverse event rate was 8.8% in the MT group vs 6.5% in the CDT group (*P* = .28), with no significant difference in the rate of thrombolysis in myocardial infarction major bleeding (1.6% vs 1.9%; *P* = .49). Moreover, 30-day mortality occurred in 30 patients (4.0%), with no statistically significant difference between the groups (4.6% vs 2.6%, *P* = .270). According to subgroup analysis, no 30-day mortality occurred in patients who initially presented as low- or intermediate-low risk. However, among intermediate-high–risk patients, 30-day mortality was similar between the treatment groups (5% vs 3%; *P* = .99). Additionally, univariate analysis revealed a significant correlation between PE severity and 30-day mortality (high risk: 9% vs intermediate-high: 3.7% vs intermediate-low: 0% vs low-risk: 0%; *P* = .022). After adjustment for age, sex, recent surgery, presenting symptoms, risk category, and malignancy, the results revealed no independent association between the type of CBT and 30-day mortality (Table III). The factors significantly associated with an increased risk of 30-day mortality included active cancer (adjusted odds ratio [OR], 4.8; 95% confidence interval [CI], 2.0-10.5; *P* < .001); systolic blood pressure (SBP) < 100 mmHg (adjusted OR, 3.4; 95% CI, 1.3-9.1; *P* < .015), and a door-to-needle time of >12 hours (adjusted OR, 3.4; 95% CI, 1.3-9.1; *P* < .015). On the other hand, syncope upon presentation was associated with a lower risk of 30-day mortality (adjusted OR, 0.22; 95% CI, 0.47-0.97; *P* < .046). The 1-year incidence rates of CTEPH and recurrent PE were 3.6% and 3.7%, respectively, with no differences between the groups (Table II). The 1-year overall survival rate was also similar between the groups (93% vs 94%; *P* > .05) (Fig).

**Table III:** Factors independently associated with 30-day mortality following catheter-based therapy for acute pulmonary embolism

Variable	Univariate analysis			Multivariate analysis		
	Unadjusted OR	95% CI	*P* value	Adjusted OR	95% CI	*P* value
Technique (MT)	1.8	0.6-5.3	.271	-	-	-
Cancer	4.3	2.0-9.3	<.001	4.81	2.2-10.5	<.001
Recent surgery	2.1	0.9-4.4	.057	-	-	-
Female sex	1.6	0.8-3.4	.20	-	-	-
Syncope	0.3	0.06-1.2	.062	0.22	0.05-0.97	.046
SBP < 100	2.3	0.9-5.9	.110	3.40	1.26-9.14	.015
Preoperative cardiac arrest	4.4	0.9-20.7	.100	11.7	2.13-64.9	.005
High risk	2.2	0.7-6.6	.138	-	-	-
DTN > 12 hours	1.9	0.8-4.4	.118	2.45	1.03-5.86	.044

*CI*, Confidence interval; *DTN*, door-to-needle time; *MT*, mechanical thrombectomy; *OR*, odds ratio; *SBP*, systolic blood pressure.

**Fig:**
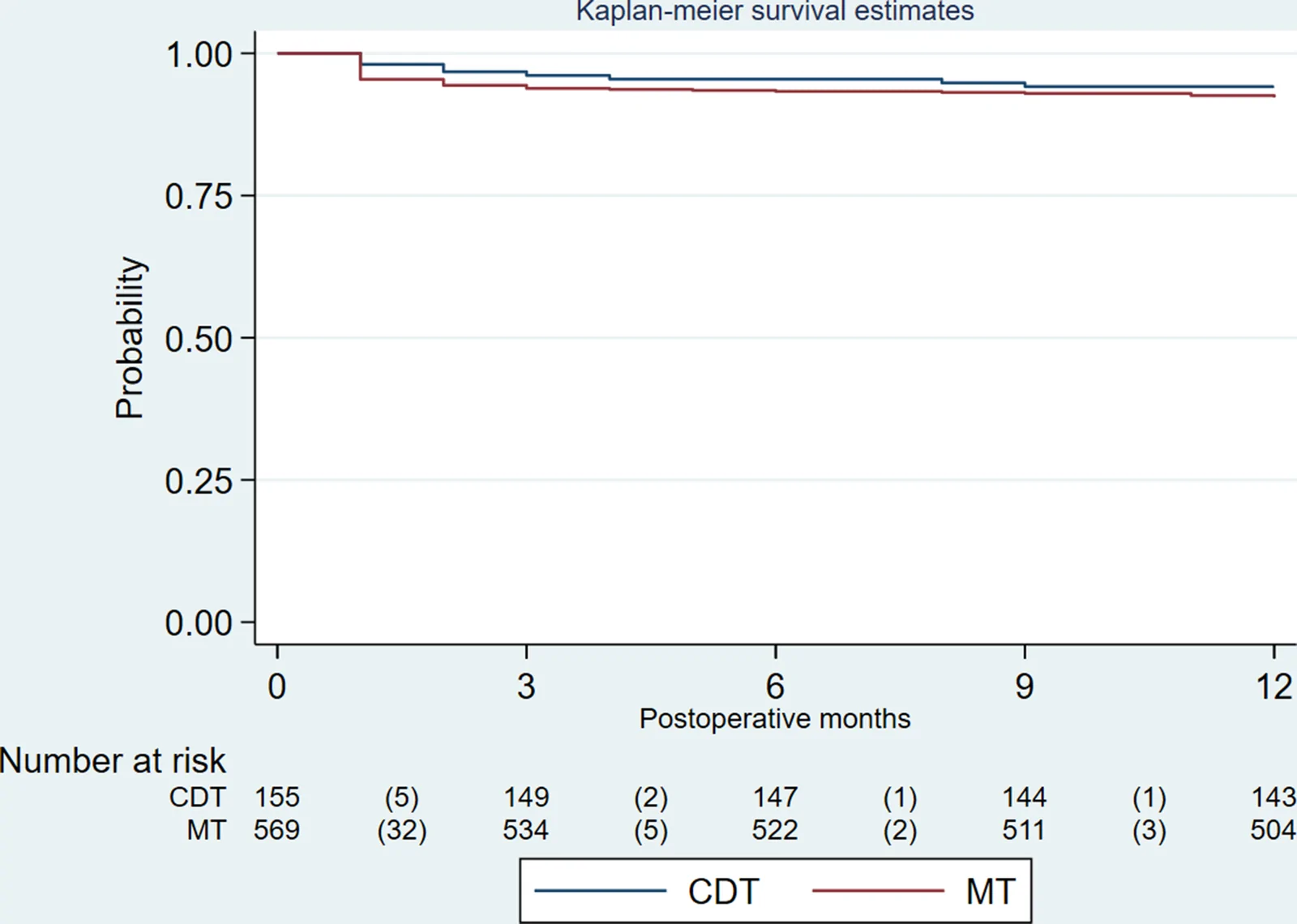
Kaplan-Meier survival estimates of 1-year freedom from all-cause mortality following catheter-based therapy for acute pulmonary embolism. *CDT*, Catheter-directed thrombolysis; *MT*, mechanical thrombectomy.

## Discussion

In this large-, single-hospital system cohort of 724 patients undergoing CBT for acute PE, we found that MT and CDT resulted in comparable short-term and 1-year clinical outcomes, including 30-day mortality, major adverse events, PE recurrence, and CTEPH development. Although differences emerged in perioperative characteristics and resource utilization, these variations did not translate into statistically significant differences in survival or long-term morbidity.

A notable observation in our cohort is that MT was preferentially performed in patients presenting with higher clinical severity, including a higher proportion of patients with high-risk PE and elevated sPESI scores. These clinical practice patterns likely reflect the institutional preference to use suction thrombectomy in patients who require rapid hemodynamic recovery or who have elevated bleeding risk from thrombolytic therapy.^13^ Despite the higher-risk profile of patients who underwent MT, 30-day mortality was not significantly different from that in patients who underwent CDT, even in the subgroup analysis of intermediate-high–risk patients. Multivariable modeling further confirmed that the treatment modality was not independently associated with 30-day mortality, whereas factors such as active malignancy, SBP < 100 mmHg, preoperative cardiac arrest, and prolonged door-to-needle time were the primary drivers of early mortality. These findings indicate that patient severity and comorbidities, rather than the choice of endovascular technique, remain the predominant determinants of early outcomes in acute PE.

The rate of periprocedural complications differed between the groups. MT was associated with higher rates of intraoperative cardiovascular collapse, a finding consistent with previous reports describing hemodynamic instability during large-bore aspiration thrombectomy.^5^, ^14^ Previous studies have proposed several potential mechanisms underlying these events. The introduction and manipulation of large-bore aspiration catheters within an already dysfunctional RV may transiently lead to the deformation of the RV or acute tricuspid regurgitation, exacerbating RV strain and impairing cardiac output, particularly in patients with severe pulmonary hypertension.^4^ Procedural complications, including direct valvular trauma and guidewire- or device-related myocardial perforation, may result in hemopericardium, with hemodynamic deterioration requiring emergent pericardial drainage or surgical intervention.^14^ Nonetheless, successful rescue and overall outcomes remain favorable, suggesting that experienced vascular teams can effectively manage these rare but critical events. Based on the intraoperative adverse cardiac events with MT that were discovered earlier on in our series, our institution subsequently adopted a treatment algorithm in which patients deemed to be at high risk of intraoperative deterioration were taken to the operating room with extracorporeal membrane oxygenation on standby before MT or CDT, if no contraindications existed.^4^ Additionally, we changed our practice to prefer smaller-bore suction thrombectomy catheters (<20F) in this subset of patients if MT was indicated.^5^, ^15^ Conversely, in the present study, CDT was associated with substantially higher ICU utilization and longer monitored postoperative care, likely reflecting institutional protocols for thrombolytic administration and monitoring requirements. The low ICU utilization rate in the MT group suggests a cost-effectiveness benefit of the treatment modality, which is outside the scope of this study and warrants further investigation. Importantly, despite these differences, the total postoperative hospital stay was similar between the groups, underscoring the overall efficiency and safety of both treatment strategies in contemporary PE response workflows.

Another interesting and unexpected finding in our study is the association between syncope at presentation and lower short-term mortality following CBT. Although the underlying mechanism is unclear, a possible explanation is that patients presenting with syncope may have been perceived to be at higher risk; consequently, they received more intensive monitoring and resuscitative measures, including ICU-level care, supplemental oxygen, and closer hemodynamic surveillance, with more timely consideration of catheter-based intervention. Such differences in early management may have contributed to improved outcomes in this subset of patients compared with patients in a similar risk category who did not present with syncope. Notably, syncope is not incorporated into commonly used PE risk stratification criteria. Further investigation is warranted to determine whether syncope independently influences the outcomes or instead serves as a marker of differences in early recognition, monitoring, and treatment intensity in patients undergoing CBT for acute PE.

At 1-year follow-up, overall survival, PE recurrence, and CTEPH incidence were low and comparable between the two treatment modalities. The relationship between the type of intervention and subsequent PE recurrence remains uncertain. Although recurrent PE is likely predominantly caused by underlying patient-specific thromboembolic risk factors, differences in the extent and distribution of thrombus clearance between CDT and MT could theoretically influence the residual pulmonary vascular obstruction and subsequent events. Some authors have demonstrated thrombus reduction in distal segmental pulmonary arteries with pharmacomechanical CDT, potentially extending treatment beyond the immediate reach of large-bore thrombectomy devices.^16^ However, whether these differences in thrombus clearance translate into reduced long-term PE recurrence remains unproven and warrants further investigation.

Our current study provides real-world, longitudinal data that add valuable insights to an evidence base that has historically been limited by short-term outcomes and selective trial populations.^10^, ^17^, ^18^, ^19^ Our findings complement the results of recent industry-sponsored trials while offering a broader view of performance across a spectrum of patient severities, comorbidities, and provider practice patterns.^19^, ^20^

### Study limitations

This study has several limitations. Its retrospective design introduces the potential for selection bias, especially regarding device choice, which was operator-dependent and likely influenced by clinical severity and operator’s expertise. Second, although this cohort study represents one of the largest single-system analyses to date, the CDT group comprised a smaller proportion of patients, which may limit comparative power for rare clinical events. Another limitation of this study is the absence of estimated blood loss as an outcome measure. We elected not to include estimated blood loss because its estimation is inherently subjective and may vary substantially across surgeons, operators, and institutions, potentially introducing measurement bias into the analysis. Changes in the hemoglobin concentration and transfusion requirements may be more objective and clinically meaningful measures of blood loss and bleeding-related morbidity. Future studies should incorporate these parameters to more comprehensively evaluate the comparative safety and effectiveness of MT and CDT. Nevertheless, the present study provides a robust assessment of real-world, system-wide practice patterns and outcomes, yielding important implications for PE response teams. As the endovascular management of PE evolves, these findings highlight that both MT and CDT are safe and effective options, with selection best tailored to patient-specific risk profiles, anatomic considerations, and institutional expertise.

## Conclusions

In this single-system retrospective study of 724 patients undergoing CBT for acute PE, MT and CDT had comparable safety and efficacy, with no statistically significant differences in 30-day mortality, major adverse events, or 1-year outcomes. MT was associated with a higher incidence of intraoperative hemodynamic complications, and CDT was linked to higher ICU utilization.

These findings support the use of MT and CDT as viable alternate treatment options within multidisciplinary PE response frameworks, with device selection optimally tailored to patient-specific risk profiles, anatomic considerations, and institutional expertise. Future prospective studies—particularly randomized trials inclusive of broader real-world populations—are needed to refine the patient selection criteria and guide the optimal use of CBTs in patients at intermediate-high-risk of PE.

## Author Contributions

Conception and design: BB, EP

Analysis and interpretation: BB, PG, SA

Data collection: BB, PG, SA

Writing the article: BB, PG

Critical revision of the article: BB, PG, SA, EP

Final approval of the article: BB, PG, SA, EP

Statistical analysis: BB

Obtained funding: Not applicable

Overall responsibility: EP

## Declaration of generative AI and AI-assisted technologies in the writing process

During the preparation of this work, the authors used Microsoft Copilot to enhance clarity and readability. After using this tool/service, the authors reviewed and edited the content as needed and take full responsibility for the content of the publication.

## Funding

None.

## Disclosures

EKP is a consultant for TVA Medical/Bard/BD Medical, Merit, Humacyte, Inc, Artegraft, Inc. The other authors declare no conflict of interest.
